# Introgression of *Swertia mussotii *gene into *Bupleurum scorzonerifolium *via somatic hybridization

**DOI:** 10.1186/1471-2229-11-71

**Published:** 2011-04-25

**Authors:** Junfeng Wang, Cuizhu Zhao, Chang Liu, Guangmin Xia, Fengning Xiang

**Affiliations:** 1 The Key Laboratory of Plant Cell Engineering and Germplasm Innovation, Ministry of Education, School of Life Sciences, Shandong University, Shanda Nanlu 27#, Jinan 250100, China; 2 Crop Germplasm Resources Centre of Shandong, Shandong Academy of Agricultural Sciences, Gongye Beilu 202#, Jinan 250100, China

## Abstract

**Background:**

The wild herb *Swertia mussotii *is a source of the anti-hepatitis compounds swertiamarin, mangiferin and gentiopicroside. Its over-exploitation has raised the priority of producing these compounds heterologously. Somatic hybridization represents a novel approach for introgressing *Swertia mussotii *genes into a less endangered species.

**Results:**

Protoplasts derived from calli of *Bupleurum scorzonerifolium *and *S. mussotii *were fused to produce 194 putative hybrid cell lines, of which three (all derived from fusions where the *S. mussotii *protoplasts were pre-treated for 30 s with UV light) later differentiated into green plants. The hybridity of the calli was confirmed by a combination of isozyme, RAPD and chromosomal analysis. The hybrid calli genomes were predominantly *B. scorzonerifolium*. GISH analysis of mitotic chromosomes confirmed that the irradiation of donor protoplasts increased the frequency of chromosome elimination and fragmentation. RFLP analysis of organellar DNA revealed that mitochondrial and chloroplast DNA of both parents coexisted and recombined in some hybrid cell lines. Some of the hybrid calli contained *SmG10H *from donor, and produced swertiamarin, mangiferin and certain volatile compounds characteristic of *S. mussotii*. The expression of *SmG10H *(geraniol 10-hydroxylase) was associated with the heterologous accumulation of swertiamarin.

**Conclusions:**

Somatic hybrids between *B. scorzonerifolium *and *S. mussotii *were obtained, hybrids selected all contained introgressed nuclear and cytoplasmic DNA from *S. mussotii*; and some produced more mangiferin than the donor itself. The introgression of *SmG10H *was necessary for the accumulation of swertiamarin.

## Background

Somatic hybridization provides a means to bypass the problem of sexual incompatibility which prevents the production of many wide hybrids in the plant kingdom. The technique has been successfully demonstrated in a number of intra- and inter-specific, intergeneric, intertribal and even inter-familial combinations [1-4]. The possibility of introgression from exotic sources is of interest not just in the applied field, but also because it provides opportunities for the discovery of novel synthetic pathways for secondary metabolites and signalling compounds.

The medicinal herb *Swertia mussotii *Franch is native to Tibet, where it has enjoyed a long history of use as a curative for hepatitis [5,6]. Its major active compounds have been shown to be swertiamarin, mangiferin and gentiopicroside [7]. The economic value of the species is such that there is now a real risk of species extinction as a result of over-exploitation. Swertiamarin and gentiopicroside are both iridoid monoterpenoids, but their synthetic pathway has not as yet been characterized in any detail [8,9]. However, many of the reactions in this pathway are known to be catalyzed by P450 proteins [10,11]. Members of this highly diverse protein family are involved in the synthesis of pigments, antioxidants and defense compounds [12], and one of particular importance for the synthesis of swertiamarin is the enzyme geraniol 10-hydroxylase (G10H) [13]. Recently, we isolated a full length cDNA clone of *S. mussotii G10H *(*SmG10H*), which has the catalytic activity of hydroxylating geraniol [14].

*Bupleurum scorzonerifolium *Willd (2n = 12), as a member of the Umbelliferae family, also is a very useful herb in Chinese traditional medicine, where it is used to treat acesodyne, diminish inflammation, ease fever and increase resistance to hepatic injury and promote immunity [15]. We previously reported plant regeneration from cultured *B. scorzonerifolium *protoplasts [16]. And these cultured cell lines with the fast-growing capacities have remained viable for at least 16 years, showing a chromosome numbers of 2n = 12 in over 90% of cells [3].

Our aim was to obtain somatic hybrids between *S. mussotii *and *B. scorzonerifolium*. The latter was chosen as the other biparent because it had a rapid growth and similar many secondary metabolic pathways [15,16]. We have used a number of fingerprinting methods to characterize the introgression events achieved by applying this process, and in particular have focussed on the presence of *SmG10H*. Finally we sought to establish the relationship between the accumulation of swertiamarin and gentiopicroside and the level of expression of *SmG10H*.

## Results

### Growth and development of somatic fusion nuclei

**Figure 1:**
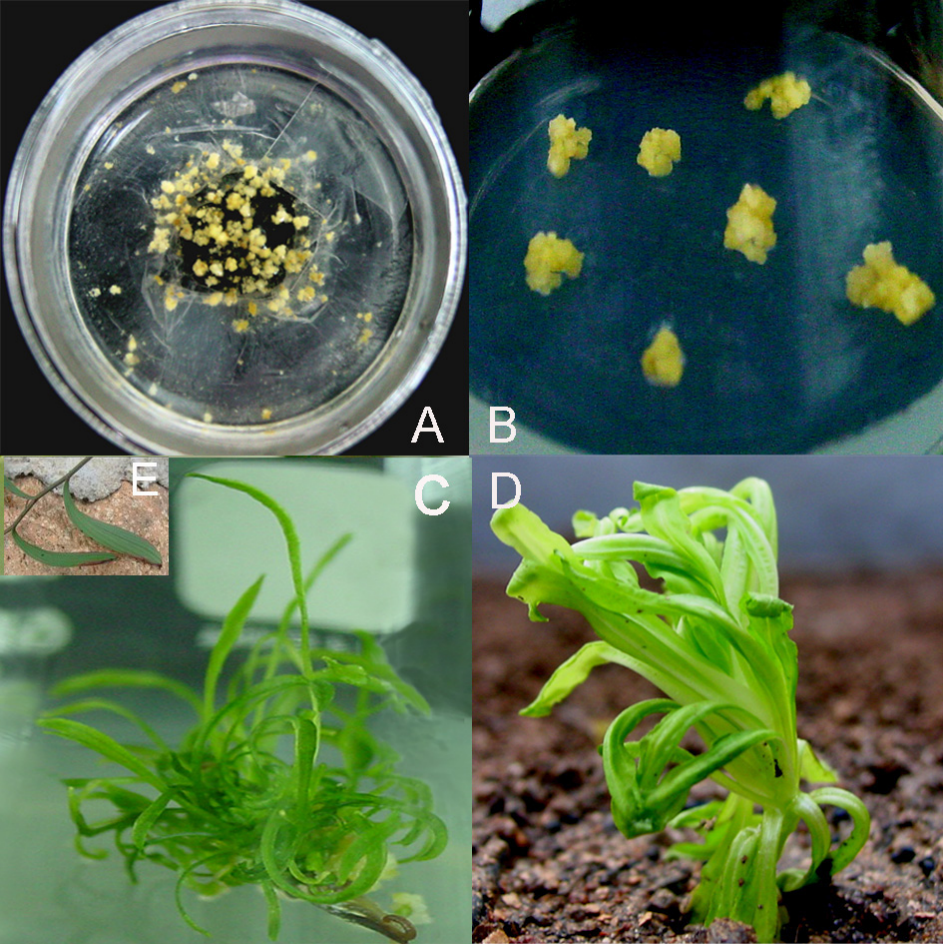
**Somatic hybridization between *B. scorzonerifolium *and *S. mussotii***. A, Calli developing 30 days after somatic hybridization; B, 60 days after somatic hybridization; C, Regenerated plant; D, *S. mussotii *plant; E, leaves of *B. scorzonerifolium*.

Granular calli were formed from combinations A-D after about two months of culture in liquid P5 medium in the dark, but no callus was observed in combination E. Once the clones had reached a diameter of 1.5-2 mm (Figure 1A), they were transferred to the proliferation medium MB_2 _(Figure 1B), where they were maintained through three rounds of sub-culturing before their final transfer to the differentiation medium MB_3_. A population of 194 clones was obtained by this process (Table 1); of these only three, all from combination B, were successfully regenerated into plants. These all developed narrow and long leaves, resembling those of *B. scorzonerifolium *(Figure 1C-E).

**Table 1:** Morphology of the biparental and hybrid calli

Combinations	Calli regeneration	Calli growth	Plant differentiation	Plant regeneration
A (UV0 s)	42 clones	fast growing	5 clones with further regeneration of shoots or roots	-
B (UV30 s)	82 clones	fast growing	14 clones with further regeneration of shoots or roots	Green plant from 3 clones
C (UV1 min)	66 clones	fast growing	7 clones with further regeneration of shoots or roots	-
D (UV2 min )	4 clones	fast growing	None	-
E (UV3 min )	-	-	-	-

### Genetic characterization of the somatic hybrid calli

**Figure 2:**
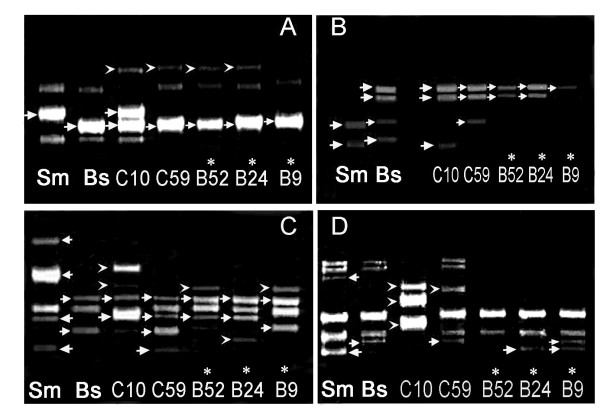
**RAPD analysis of hybrid clones**. A, Primer H19; B, Primer Q15; C, Primer Q8; D, Primer N20. Sm, *S. mussotii*; Bs, *B. scorzonerifolium*. Lanes 2, 3, 10, 13 and 14 refer, respectively to hybrid clones B9, B24, B52, C59 and C10. **→**, Distinctive bands inherited from the donor or recipient. ►, Bands not present in either the donor or the recipient. *, The regenerated hybrid clones.

Esterase isozyme analysis of 194 clones indicated that 104 had the partial characteristic band(s) of both parents and novel bands and were verifiable as hybrid (Additional file 1). A set of 88 RAPD primers was applied to generate DNA fingerprints of the presumptive hybrid calli (Additional file 2). Fragments from both biparents, as well as fragments not present in either of them, were observed in all of the hybrid clones tested (Figure 2). As 91% of the fragments in the hybrids were derived from the *B. scorzonerifolium *biparent, and only 0.2-2.4% from the *S. mussotii *biparent, the hybrid genomes were dominated by the recipient species (Table 2). The construction of donor nuclear genome DNAs in these hybrids were similar except hybrid C10 (Additional file 3). RAPD analysis showed in hybrids exposure of UV for 30 s, there were 1.7% donor characteristic bands and 1.2% new bands (2.9% in total), however the numbers were raised to 4.5% and 3.2% (7.7% in total) in hybrids exposure of UV for 1 min (Additional file 3).

**Table 2:** Frequency of donor and recipient RAPD DNA fragments among hybrid calli and regenerated plants

	Total bands	Characteristic bands		Frequency (%)	
		
		Acceptor bands	Donor bands	Acceptor bands/Total bands	Donor bands/Total bands
Hybrid B9	538	524	3	96.68	0.60
Hybrid B24	527	523	1	97.48	0.20
Hybrid B52	526	517	4	96.46	0.81
Hybrid C59	528	521	2	97.02	0.41
Hybrid C10	539	498	12	91.80	2.41
*B. scorzonerifolium*	546	-	-	-	-
*S. mussotii*	456	-	-	-	-
Average		516.6 (± 10.7)	4.4 (± 4.4)	95.9 (± 2.3)	0.9 (± 0.9)

### Karyotypes of somatic hybrid clones

**Figure 3:**
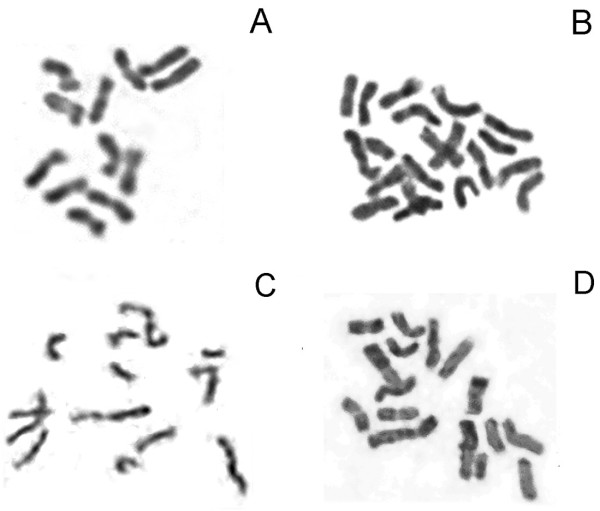
**Mitotic chromosome numbers in hybrid clones**. A, *B. scorzonerifolium *2n = 12; B, *S. mussotii *2n = 20; C, Hybrid clone C10 2n = 13; D, Hybrid clone B24 2n = 16.

**Figure 4:**
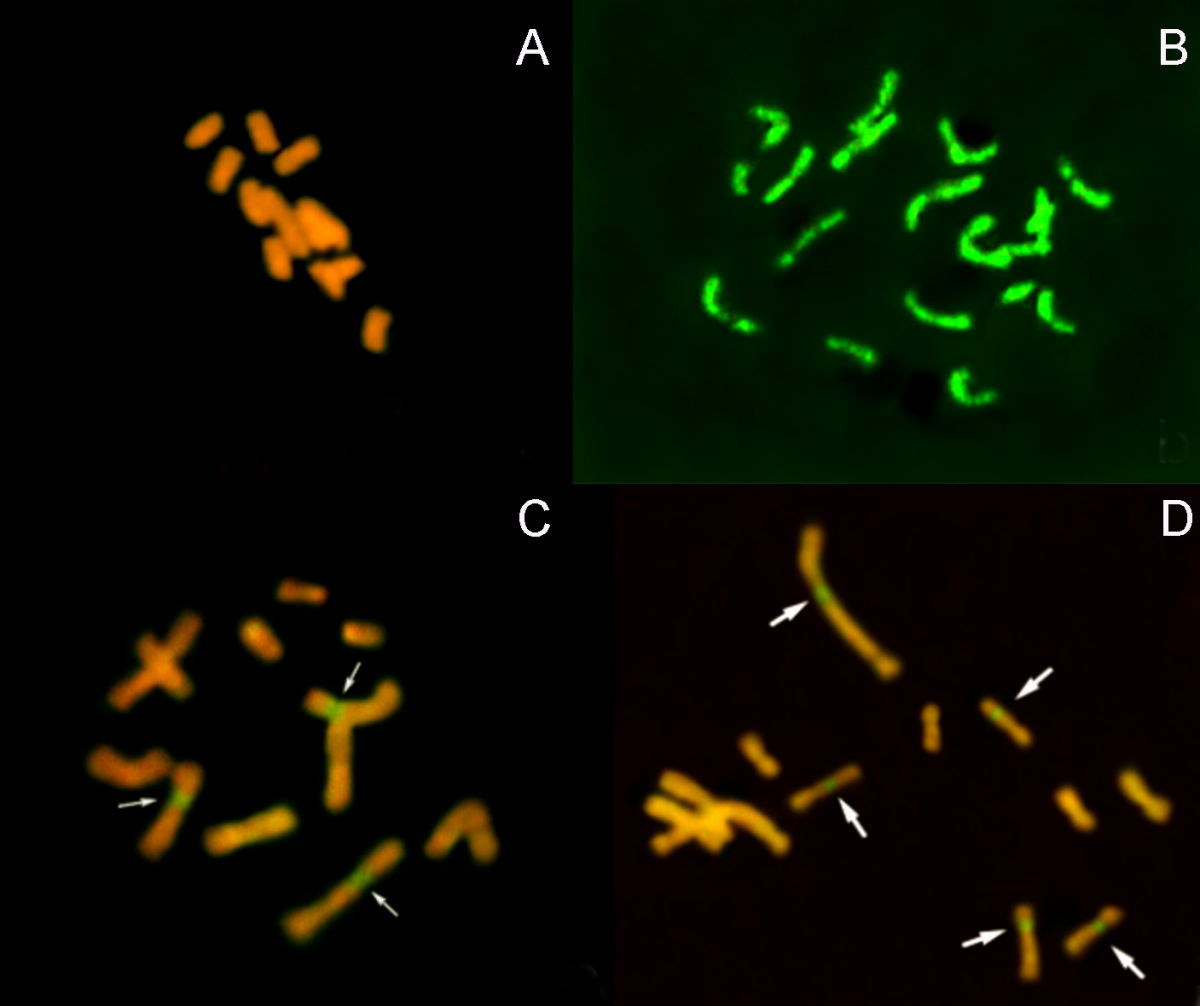
**GISH analysis of mitotic chromosomes in hybrid clones**. A, *B. scorzonerifolium*; B, *S. mussotii*; C, Hybrid clone B24; D, Hybrid clone C10. →, Presence of donor chromosome segment.

The chromosome numbers of *B. scorzonerifolium *and *S. mussotii *calli were 11-12 and 17-20, respectively (Figure 3A, B and Table 3). In the clones derived from combination A, the number was no lower than 15, with most carrying 17-20 (Table 3). Combination B clones carried 11-16 chromosomes, and combination C ones carried 11-14 (Table 3). Combination D clones had 11-14 (Table 3). When analysed using GISH, the biparental genomes were readily distinguishable from one another (Figure 4A, B). The three regenerable hybrid clones had chimera cells with different chromosome numbers, carrying 11-13 intact *B. scorzonerifolium*, none intact *S. mussotii*, and 1-3 recombined chromosomes (Figure 4C). In contrast, the non-regenerable clones carried 11-13 intact *B. scorzonerifolium*, none intact *S. mussotii *and 5-9 recombined chromosomes (Figure 4D).

**Table 3:** Variation for somatic chromosome number in biparental and hybrid calli

	Number of cell samples	Numeber of chromosomes				
		
		11-12	13-14	15-16	17-18	19-20
*B. scorzonerifolium*	33	33	-	-	-	-
*S. mussotii*	42	-	-	-	7	35
Hybrid A10	37	-	-	7	25	5
Hybrid A22	36	-	-	4	23	9
Hybrid A24	61	-	-	12	41	8
Hybrid B9	59	-	7	39	13	-
Hybrid B24	63	-	41	10	12	-
Hybrid B52	49	-	5	36	8	-
Hybrid C10	51	13	38	-	-	-
Hybrid C64	44	12	32	-	-	-
Hybrid C88	62	22	40	-	-	-
Hybrid D2	34	23	11	-	-	-

### Analysis of the cytoplasmic genomes of somatic hybrids

**Figure 5:**
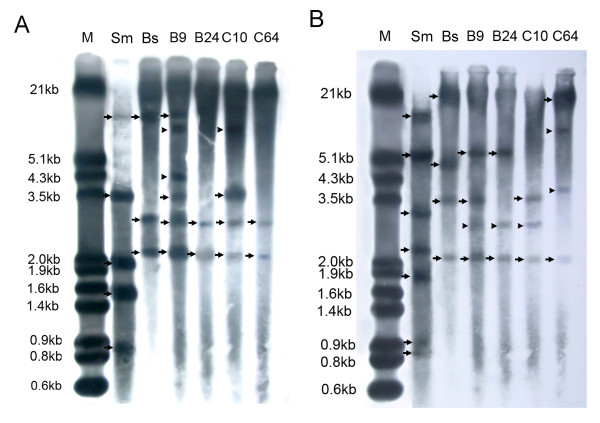
**RFLP of profiles of mitochondrial and chloroplast DNA of *S. mussotii, B. scorzonerifolium*, and the hybrid cell lines from combinations B and C between *S. mussotii *and *B. scorzonerifolium***. M, labeled λDNA digested by HindIII+EcoRI; Bs, *B. scorzonerifolium*; Sm, *S. mussotii*; B9, B24, C10 and C64, hybrid cell lines of *S. mussotii -B. scorzonerifolium*. Arrows indicate bands of the *S. mussotii *and *B. scorzonerifolium*; arrowheads indicate new bands. A, HindIII-digested genomic DNA probed with the mitochondrial-specific probes coxI. B, HindIII-digested genomic DNA probed with the chloroplast-specific probe rbcL.

The RFLP profiles of mitochondrial DNA obtained using restriction enzymes HindIII and hybridized with probes coxI revealed that all of the cell lines analyzed contained *B. scorzonerifolium *sequences and cell lines B9 and C10 had donor bands and novel bands (Figure 5A). The chloroplast type of the hybrid cell lines was determined using rbcL as a hybridization probe. Hybridizations of HindIII digests with rbcL show that all of the cell lines analyzed contained *B. scorzonerifolium *fragments and 1-2 novel fragments, and cell lines B9 and B24 contained *S. mussotii *fragments (Figure 5B). Thus, some recombination within the mitochondrial and chloroplast genome of both parents also occurred in some hybrids.

### Content of medicinally active compounds

**Figure 6:**
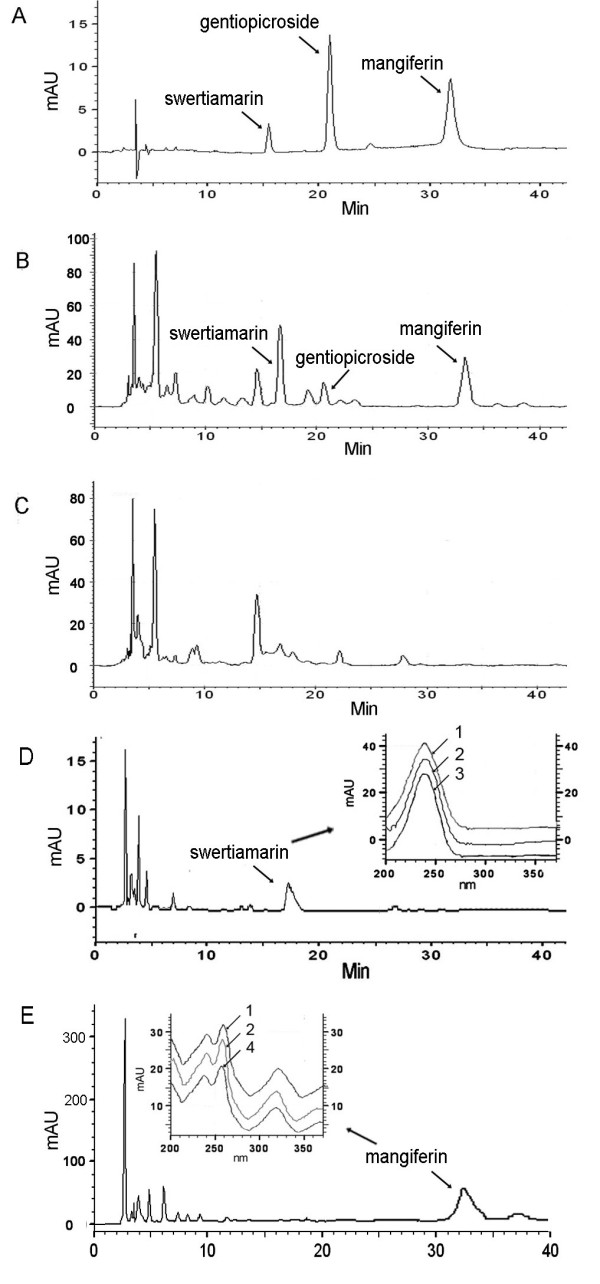
**HPLC analysis of hybrid clones**. A, Standard preparations of swertiamarin, gentiopicroside and mangiferin; B, *S. mussotii*; C, *B. scorzonerifolium*; D, Hybrid clone B24; E, Hybrid clone C18. UV spectrum of swertiamarin and mangiferin from samples were indicated in D and E. 1, *S. mussotii*; 2, Standard preparations; 3, Hybrid clone B24; 4, Hybrid clone C18.

The HPLC-based analysis of 74 of the hybrid clones determined that none accumulated gentiopicroside (Figure 6). Clones B24, B27, B132, C18, C26, C47 and C124 contained 7.4-81.2 μg/g swertiamarin, while clones B6, B40, B56, C10 and C121 contained 86.3-816.8 μg/g mangiferin. Notably, the mangiferin content of clones B6, B56 and C10 was higher than that of the callus derived from the *S. mussotii *biparent (Table 4).

**Table 4:** Content of medicinally active compounds in biparental and hybrid calli

	Swertiamarin		Gentiopicroside.		Mangiferin	
	
	Retention time (min)	Content (mg/g)	Retention time (min)	Content (mg/g)	Retention time (min)	Content (mg/g)
*S. mussotii*	16.93 ± 0.10	0.9298 ± 0.0039	20.65 ± 0.17	0.4235 ± 0.0026	33.51 ± 0.14	0.6641 ± 0.0027
*B. scorzonerifolium*	-	-	-	-	-	-
Hybrid A6	-	-	-	-	-	-
Hybrid A67	-	-	-	-	-	-
Hybrid B6	-	-	-	-	31.75 ± 0.15	0.7970 ± 0.0034
Hybrid B24	17.55 ± 0.15	0.0812 ± 0.0022	-	-	-	-
Hybrid B27	17.05 ± 0.14	0.0769 ± 0.0026	-	-	-	-
Hybrid B40	-	-	-	-	31.37 ± 0.16	0.0996 ± 0.0033
Hybrid B56	-	-	-	-	31.79 ± 0.12	0.7903 ± 0.0029
Hybrid B132	16.43 ± 0.16	0.0746 ± 0.0023	-	-	-	-
Hybrid C10	-	-	-	-	32.24 ± 0.13	0.8168 ± 0.0037
Hybrid C18	16.87 ± 0.12	0.0083 ± 0.0015	-	-	-	-
Hybrid C26	16.68 ± 0.13	0.0082 ± 0.0012	-	-	-	-
Hybrid C47	16.29 ± 0.14	0.0102 ± 0.0017	-	-	-	-
Hybrid C121	-	-	-	-	32.08 ± 0.14	0.0863 ± 0.0034
Hybrid C124	17.21 ± 0.18	0.0074 ± 0.0016	-	-	-	-

### Volatile compounds in the biparent and hybrid clones

The volatile compound content of the hybrid clones, as assessed by GC-MS, largely resembled that of the *B. scorzonerifolium *biparent (Additional file 4). Nevertheless, a few donor compounds, in particular coumaron and linoleic acid, were detectable in some of the hybrid clones, along with a small number of compounds (e.g., cyclohexanol and dodecanoyl) which were not detected in either biparent (Table 5).

**Table 5:** Partial special volatile compounds in hybrids compared with the parents

Sample	Compound	Molecular formula	*S. mussotii*	Hybrid A6	Hybrid B24	*B. scorzonerifolium*
1	2-pyrrolidone	C_4_H_7_NO	-	-	+	+
2	dihydropyran	C_5_H_8_O	+	-	+	-
3	2,3 -dimethyl pyrazine	C_6_H_8_N_2_	+	-	+	-
4	cyclohexanol	C_6_H_12_O	-	+	+	-
5	2,4-dihydroxy-2,5-dimethyl-3(2H)-furanone	C_6_H_8_O_4_	+	+	-	-
6	2,5- methenyl furfuran	C_6_H_3_O_3_	-	+	+	-
7	2,5-dimethyl-4-hydroxy-3(2H)-furanone	C_6_H_8_O_3_	-	+	-	-
8	ethyl vinyl ether	C_6_H_12_O	-	-	+	-
9	phenol	C_6_H_6_O	+	+	+	-
10	heptylic acid	C_7_H_14_O_2_	+	-	+	-
11	diethyl cyclopentane	C_7_H_20_	-	+	+	+
12	1-(3-aminopropoxy)-2-ethoxyethane	C_7_H_17_NO_2_	+	-	+	-
13	salicylic acid	C_7_H_6_O_3_	+	+	+	-
14	hyacinthin	C_8_H_8_O	-	-	+	+
15	2,5-dimethyl-4-hydroxy-3-furanone	C_8_H_14_O_3_	-	+	-	+
16	benzendicarboxylic acid	C_8_H_6_O_4_	+	-	+	-
17	coumaron	C_8_H_8_O	+	-	+	-
18	methylnaphthalene	C_11_H_10_	-	-	+	+
19	dodecanoyl	C_12_H_24_O	-	+	+	-
20	tetradecanoic acid	C_14_H_28_O_2_	-	+	-	+
21	tetradecanol	C_14_H_28_O	+	-	+	+
22	1-hydroxy-3,7,8-trimethoxyxanthenone	C_16_H_14_O_6_	+	-	+	-
23	linoleic acid	C_18_H_32_O_2_	+	-	+	-
24	1,2 benzendicarboxylic acid octyl nitrite	C_24_H_38_O_4_	+	+	-	-
25	2-ethylhexyl hexadecanoate	C_24_H_48_O_2_	+	+	-	+

### The introgression of P450 genes

**Figure 7:**
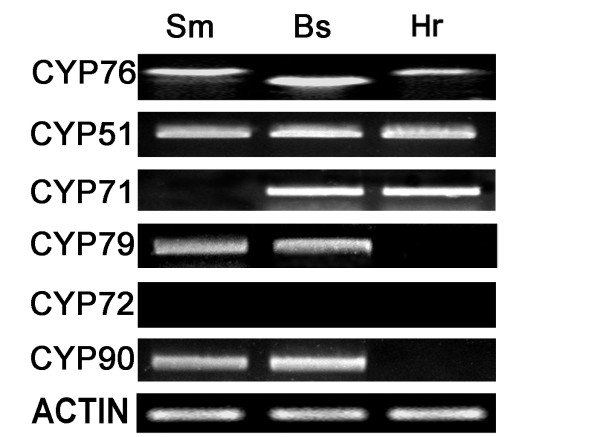
**Allelic variation for G10H in hybrid and biparent calli**. Sm, *S. mussotii*; Bs, *B. scorzonerifolium*; Hr, hybrid.

Degenerate PCR analysis was used to detect the P450 genes in clones A6, A67, B24, B27, B132, C18, C26, C47 and C124, with various contents of swertiamarin and mangiferin (Figure 7 and Additional file 5). Only amplicon of primer CYP76 was distinguished among the bipatents and hybrid A6 (Figure 7). Each cDNA template amplified a single fragment in the size range 1100~1500 bp in hybrids above and the bipatents using primer CYP76. Sequencing identified 11 distinct fragments. An analysis of the set of polypeptides predicted from these nucleotide sequences identified their homology to the G10H gene of *Catharanthus roseus *(geraniol 10-hydroxylase gene, GenBank accession number AJ251269). A full length *SmG10H *sequence of 1488 bp (Genebank accession GU168041) was obtained from *S. mussotii*. The *G10H *sequences present in clones B24, B27, B132, C18, C26, C47 and C124 were identical to that of *SmG10H *(Additional file 6). In two clones (A6 and A67), the *G10H *sequence shared 53.1% homology with *SmG10H *(Additional file 7).

### Up-regulation of SmG10H is correlated with the accumulation of swertiamarin

**Figure 8:**
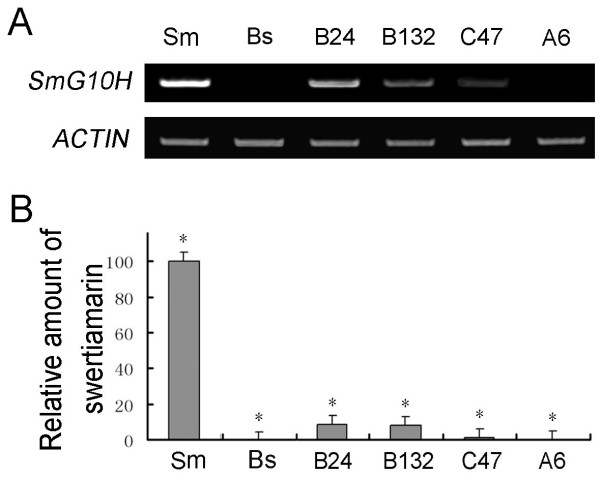
**The expression of SmG10H and accumulation of swertiamarin in hybrid clones**. A, Variation for level of *SmG10H *expression. B, Swertiamarin content. Sm, *S. mussotii*; Bs, *B. scorzonerifolium*; B24 and B132, hybrid clones from combination B; C47, hybrid clone from combination C; A6, hybrid clone from combine A. Bars represent the standard error of the mean; *t *test, *, P < 0.05.

Semi-quantitative RT-PCR suggested that the expression *SmG10H *varied among the clones (*S. mussotii *> B24 > B132 > C47 > A6, see Figure 8 and Table 4). The swertiamarin content of *S. mussotii *(933 μg/g) was substantially higher than that in the hybrid clones (0-81.2 μg/g), while clones B24 and B132 produced more than clone C47; neither swertiamarin nor *SmG10H *expression were detected in clone A6. These results suggest that up-regulation of SmG10H is correlated with the accumulation of swertiamarin.

## Discussion

### Hybrid clones experience both chromosome elimination and introgression

Across a range of hybrid combinations, the regeneration of viable plants has proven to be the main bottleneck in the somatic hybridization process [1,3,4]. Much of the problem appears to be related to the hybrid incompatibility of the biparents. This hybrid incompatibility can be alleviated if sufficient of the donor biparent's chromosomes are either completely eliminated, or at least are broken down so that sub-chromosomal segments become fused with the recipient biparent's chromosome [2,17,18]. The somatic chromosome number of successful regenerants has been shown to be close to or just slightly lower than that of the recipient biparent [19,20]. Here, only three of the population of the 194 somatic *B. scorzonerifolium */ *S. mussotii *fusion nuclei proved to be regenerable. Both the genetic and cytological analyses showed that the constitution of the regenerable hybrid calli was close to that of the recipient parent *B. scorzonerifolium*, which suggested that large-scale chromosome elimination is necessary to restore the somatic hybrids' ability to regenerate.

UV irradiation of the donor biparent's protoplasts prior to fusion has been shown to encourage chromosomal elimination [21-23]. The hybrid cell lines B24 and C10 both retained 11-13 *B. scorzonerifolium *chromosomes, none entire *S. mussotii*, but the former retained 1-3 introgression chromosomes, while the latter retained more (5-9) introgression chromosomes (Figure 4). This result is consistent with the pattern whereby raising the UV dosage decreases the number of intact donor chromosomes but increases the frequency of donor introgression [20,23].

### Characteristics of the hybrid cytoplasmic genome

Earlier investigations showed that recombination and (or) coexistence mitochondria DNA from both parents is common in somatic hybrids [23]. In contrast, chloroplast DNA often had random and equal segregation [24,25]. Mixed populations and recombination of chloroplast DNA have only rarely been detected [26]. In our previous studies, mixed and recombined mitochondrial DNA was also seen in wheat somatic hybrids [23,27,28]. In this study, the mitochondrial and chloroplast DNA of both parents also coexisted in most hybrid cell lines. Novel DNA segments appeared in some hybrids, which may have been the result of recombination of mitochondria and chloroplast DNA (Figure 5). We conclude that in the inter-familial hybridization between *B. scorzonerifolium *and *S. mussotii*, it is possible to transfer donor mitochondrial and chloroplast genes.

### The engineering of medicinally active compounds

Somatic hybrids could increase the content of the efficacious compounds from traditional Chinese medicinal materials. However, only a few cases succeeded [29]. The content of swertiamarin, mangiferin and gentiopicroside varied markedly among the hybrid clones (Table 4). With respect to swertiamarin, no clone accumulated close to the level which was achieved by the donor calli (Figure 6). However, with respect to mangiferin, five clones (B6, B40, B56, C10 and C121) outperformed the donor, two accumulated markedly less (B40 and C121), and two (A6 and B24) produced no detectable level (Table 4). The accumulation by the hybrid clones of a number of volatiles associated with the donor species is also indicative of the transfer of whole synthetic pathways from *S. mussotii *to a genotype which is largely *B. scorzonerifolium*.

The clones best able to accumulate mangiferin tended to have retained the most introgressed chromosomes (Figure 4). Similarly the RAPD fingerprinting showed that these clones also inherited the most donor DNA (Table 2 and Figure 2). Presumably maximizing the yield of the donor's medicinally active compounds in a somatic hybrid clone requires transferring as much donor DNA as possible.

### The relationship between *SmG10H *expression and swertiamarin content

According to RT-PCR at least, the expression of *SmG10H *varied among the hybrid clones. Nevertheless, its level was largely correlated with the accumulation of swertiamarin (Figure 8), implying that the transfer of *SmG10H *alone cannot be expected to be sufficient to guarantee heterologous expression. The implied requirement for other genes in the synthetic pathway underlines the difficulty that a more reductive, tansgenic strategy would face in obtaining the successful production of swertiamarin in a heterologous situation. The identification of hybrid clones able to accumulate this medicinally significant compound therefore confirms the potential of somatic hybridization as a viable route for engineering the production of such molecules in plants.

## Conclusions

In conclusion, somatic hybridization provides a new way to introgression secondary metabolites and related genes in phylogenetic distant species. Here we have managed to obtain somatic hybrids of *B. scorzonerifolium / S. mussotii *with an appreciable content of swertiamarin. The nuclear and cytoplasmic genes from donor were transferred into the genome DNA of hybrid clones. The introgression of *SmG10H *was necessary for the accumulation of swertiamarin. Therefore, the potential of somatic hybridization is a viable route for engineering the production of such molecules in plants.

## Methods

### Origin of biparental protoplasts

Immature seed of *Swertia mussotii *Franch was collected from Yushu county, Qinghai province, China. Voucher specimens had been deposited at Qinghai Normal University. The seed was surface-sterilized by immersion first in 70% (v/v) ethanol for 30 s and then in 0.1% w/v aqueous mercuric chloride for 10 min. The seeds were plated on Murashige and Skoog [30] basal medium (MS) containing 1 mg/l 2, 4-dichlorophenoxyacetic acid (2, 4-D) using the method described by Xiang *et al. *[31] to induce the production of callus, the source of donor protoplasts. The callus was subcultured on MB medium (MS medium supplemented by B5 vitamins [23], 2 mg/l glycine, 146 mg/l glutamine, 300 mg/l casein hydrolysate, 1 mg/l 2, 4-D, 30 g/l sucrose and 7.5 g/l agar at pH 5.8) at interval of 15 days. The recipient protoplasts were obtained from *Bupleurum scorzonerifolium *Willd calli induced in the same way; these have been kept in culture for 12 years under 18-20 μmol m^-2 ^s^-1 ^cool white light in MB medium. Protoplasts were isolated from all calli following established methods [32].

### Protoplast fusion and post fusion culture

Preparations of both protoplast types were washed in 0.6 M mannitol, 5 mM CaCl_2_, then transferred to a 3.5 cm petridish to form a thin layer. Donor protoplasts were UV irradiated at 380 μW/cm^2 ^for either 0 s (S1), 30 s (S2), 1 min (S3), 2 min (S4) or 3 min (S5), after which they were mixed with the recipient protoplasts at a ratio of 1:1. The fusion protocol followed the PEG method described by Xia and Chen [32]. Fusion products, which combinations A-E were corresponding donor protoplasts S1-S5, were cultured on P5 medium [32]. Once calli had reached a diameter of 1.5-2.0 mm, they were transferred to MB_2 _medium and sub-cultured every two weeks for 1-2 months. At this stage, the calli were removed to a MB_3 _solid medium (MB medium supplemented with 1 mg/l 6-benzylaminopurine and 1 mg/l indoleacetic acid). Regenerated plantlets were transferred to a seedling-strengthening medium [32].

### Callus genotying

Esterase isozymes were extracted from calli and assayed as described elsewhere [33]. DNA was extracted from selected calli according to Doyle and Doyle [34], and used as template for RAPD reactions based on 88 decamer primers (Promega Inc., Madison, Wis.). The PCRs were conducted according to Xia *et al. *[35], and the amplicons were electrophoresed through 1.5% agarose gels before staining in 0.5 μg/ml ethidium bromide. These RAPD experiments were repeated at least 3 times and only the repeatable bands were record.

### Mitotic chromosome analysis

Mitotic chromosome spreads from callus and root tip cells were prepared as described by [20]. For GISH analysis, total genomic *S. mussotii *DNA was used as the probe, and the procedure described by Xiang *et al. *[20] was applied.

### RFLP analyis

Genomic DNA of the putative hybrid cell lines and their parents was extracted as described previously [34]. For the analysis of chloroplast (cp) and mitochondrial (mt) DNA, 15-20 μg of total DNA was digested with HindIII and electrophoresed through 0.8-1% agarose gels in TBE buffer. The DNA was transferred onto a nylon membrane (Hybond N+, Amersham-Phamarcia, UK) using 0.4 M NaOH. Probe labelling, hybridization, and washing were carried out with the ECL Random Labeling and detection system (Amersham-Phamarcia, UK) according to the manufacturer's instructions. Plasmids containing mtDNA fragments (coxI) from maize (*Zea mays *L.) and a cpDNA fragment (rbcL) from spinach (*Spinacia oleracea *L.) were kindly provided by Dr. G. Spangenberg (Institute for Plant Sciences, Swiss Federal Institute of Technologies, CH-8092 Zürich, Switzerland). The inserts were cut out of a gel and labelled.

### Semi-quantitative RT-PCR analysis

Semi-quantitative RT-PCR was conducted on total RNA isolated from hybrid and two parents calli using the TriZOL reagent (Invitrogen, USA). First strand cDNA was synthesized using Superscript II reverse transcriptase M-MLV (TakaRa, Japan), following the manufacturer's directions. Degenerate primers targeting the gene from *Arabidopsis thaliana *encoding cytochrome P450 monooxygenase (Additional file 2) were applied to the cDNA templates. The amplicons derived from degenerate primers targeting the two *S. mussotii *actin genes *SmAct1 *and *SmAct2 *were used to normalize the RT-PCR signal. Each PCR comprised 19-25 cycles of 94°C/30 s, 53°C/30 s, 72°C/90 s, and was completed with a 10 min extension at 72°C. Each PCR was replicated at least three times, based on independent biological samples.

### HPLC analysis

The calli were shade-dried for seven days and ground to powder. After the addition of 20 ml methanol to 1 g powdered callus, the resulting suspension was sonicated for 1 h at room temperature, then filtered through a 0.45 μm membrane filter. A 10 μl aliquot of filtrate was injected into a LC-10AD HPLC system (Shimadzu Co., Japan) equipped with a C18 column (Phenomenex Luna, 4.6 × 250 mm i.d., 5 μm). The mobile phase was water:methanol (75:25), and the outflow (0.8 ml/min) was scanned at 259 nm [14]. Standards for swertiamarin, gentiopicroside and mangiferin were provided by the National Institute for the Control of Pharmaceutic and Biological Products (Beijing, China). All above experiments were carried out four times. In all statistical tests, values of P lower than 0.05 were interpreted as indicating statistically significant differences. Results were analysed with SAS statistical package (Version5.1, SAS Institute Inc., Cary, NC).

### Capillary gas chromatography/mass spectrometry (GC-MS) analysis

Methanol extracts of the calli were prepared according to Liu *et al. *[36], and then subjected to GC/MS, using a Micromass GCT gas chromatograph-mass spectrometer (England) fitted with a DB-5 ms column (0.25 mm × 30 m, 0.25 μm film thickness) (J W Scientific, Folsom, CA), with a helium flow rate of 1 ml/min, and operating at 70 eV ionization voltage with a scan range of 20-600 Da. The column temperature was set at 200°C for 2 min, then elevated to 300°C at 15°C/min and held at 300°C for 7 min.

## Authors' contributions

JFW conducted most of the experiments and helped in the writing of the ms; CZZ contributed to the GC/MS experiment and participated in the drafting of the manuscript; GMX was responsible for the design and coordination of the study; FNX conceived the study and was responsible for the final version of the ms. The final manuscript were read and approved by all authors.

## Supplementary Material

Additional file 1 **Esterase analysis of calli**. Sm, *S. mussotii*; Bs, *B. scorzonerifolium*. ►, Isozymes not present in either the donor or the recipient; **→**, Distinctive isozymes inherited from the donor or recipient. *, important calli. Click here for file

Additional file 2 **RAPD analysis of biparental and hybrid calli**. Sm: Fragments inherited from *S. mussotii*; Bs: Fragments inherited from *B. scorzonerifolium*; T, total of the parents and new bands; N: fragments not present in either biparental profile. Click here for file

Additional file 3 **Frequency in the hybrid clones of donor fragments, and fragments absent from both biparental profiles**. Click here for file

Additional file 4 **GC-MS analysis of volatile compounds present in the biparental and hybrid calli**. Click here for file

Additional file 5 **Sequences of primer used for these experiments**. Click here for file

Additional file 6 **Alignment of G10H nucleotide sequences**. Smg10 h, g10 h from *S. mussotii*; B24 g10 h, g10 h from hybrid B24; C26 g10 h, g10 h from hybrid C26. Click here for file

Additional file 7 **Alignment of G10H peptide sequences**. The red line indicates the conserved domain within the sequence. SmG10H-P, G10H from *S. mussotii*; B24G10H-P, G10H from hybrid B24; A6G10H-P, G10H from hybrid A6; BsG10H-P, G10H from *B. scorzonerifolium*. Click here for file
