# Supplementary figures and images for: Introgression of Swertia mussotii gene into Bupleurum scorzonerifolium via somatic hybridization
Source: BMC Plant Biol. 2011 Apr 25;11:71. doi: 10.1186/1471-2229-11-71 (PMC3098146; doi:10.1186/1471-2229-11-71)

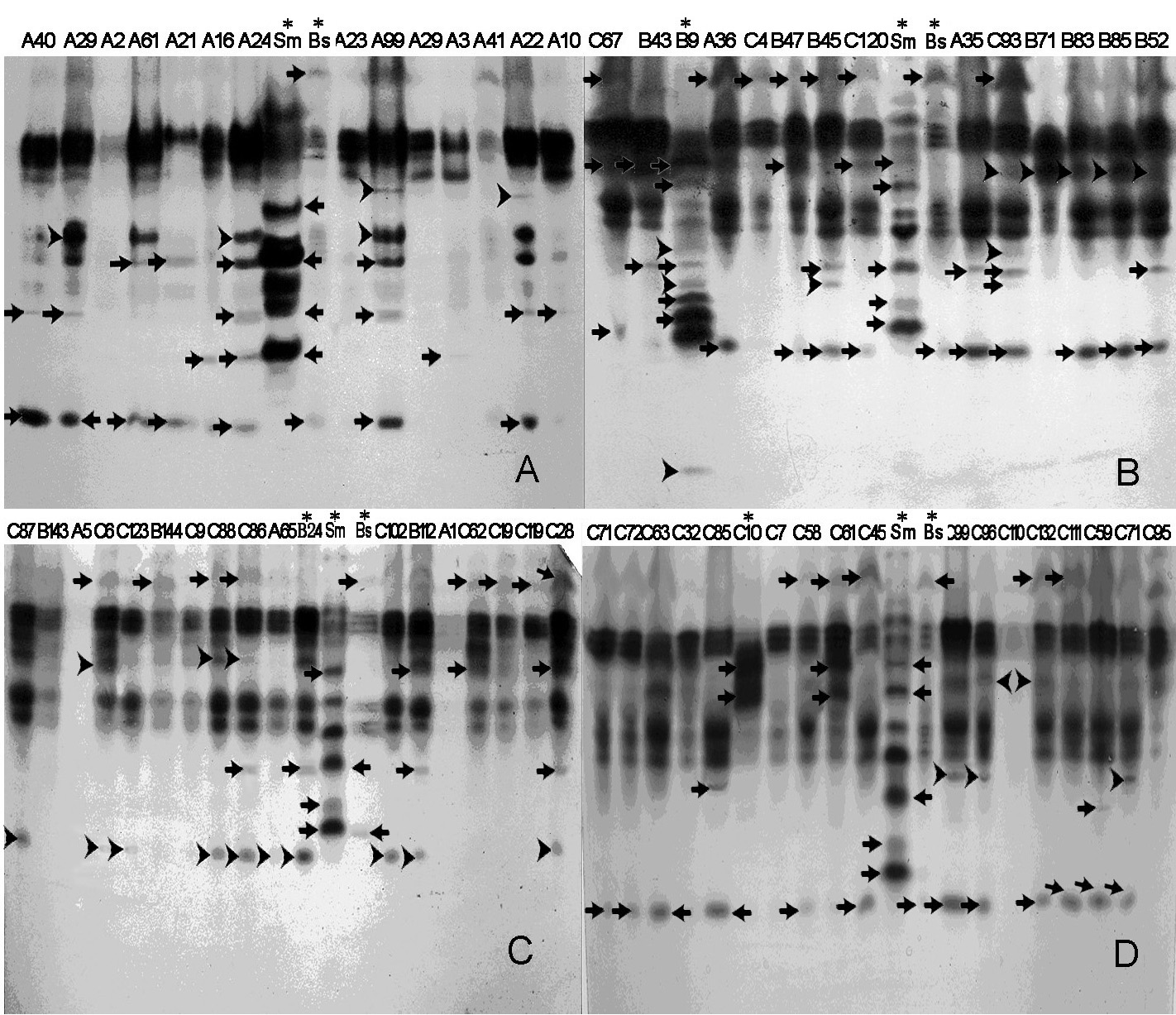

Supplement: Additional file 1 — Esterase analysis of calli. Sm, S. mussotii; Bs, B. scorzonerifolium. ►, Isozymes not present in either the donor or the recipient; →, Distinctive isozymes inherited from the donor or recipient. *, important calli. [file 1471-2229-11-71-S1.JPEG]

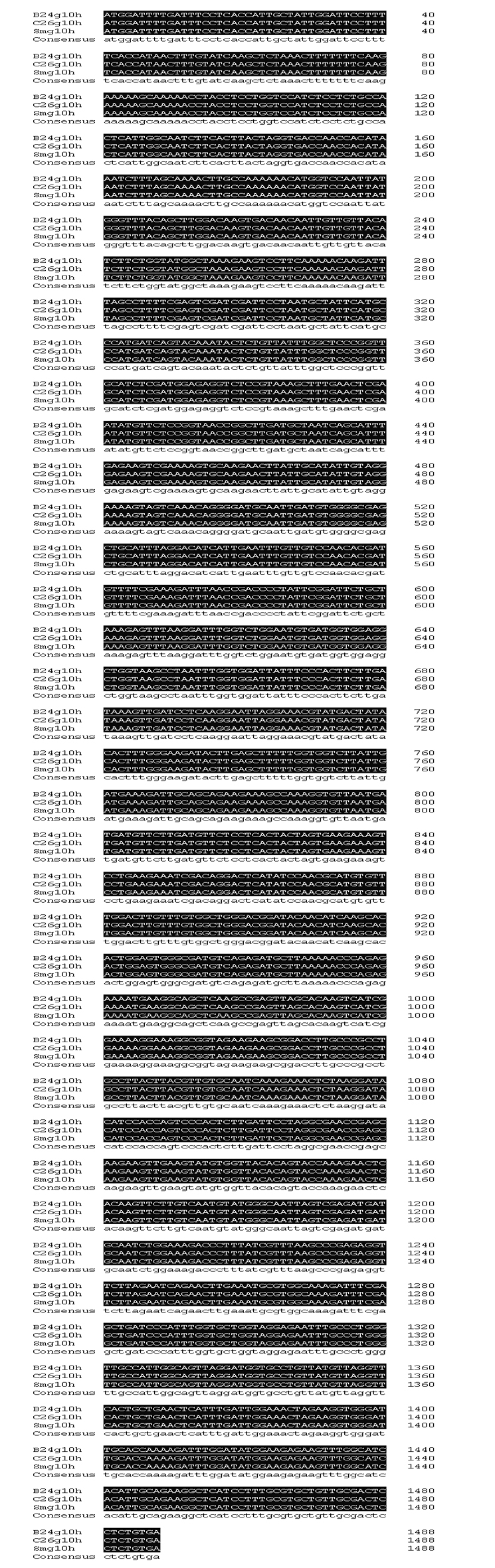

Supplement: Additional file 6 — Alignment of G10H nucleotide sequences. Smg10 h, g10 h from S. mussotii; B24 g10 h, g10 h from hybrid B24; C26 g10 h, g10 h from hybrid C26. [file 1471-2229-11-71-S6.JPEG]

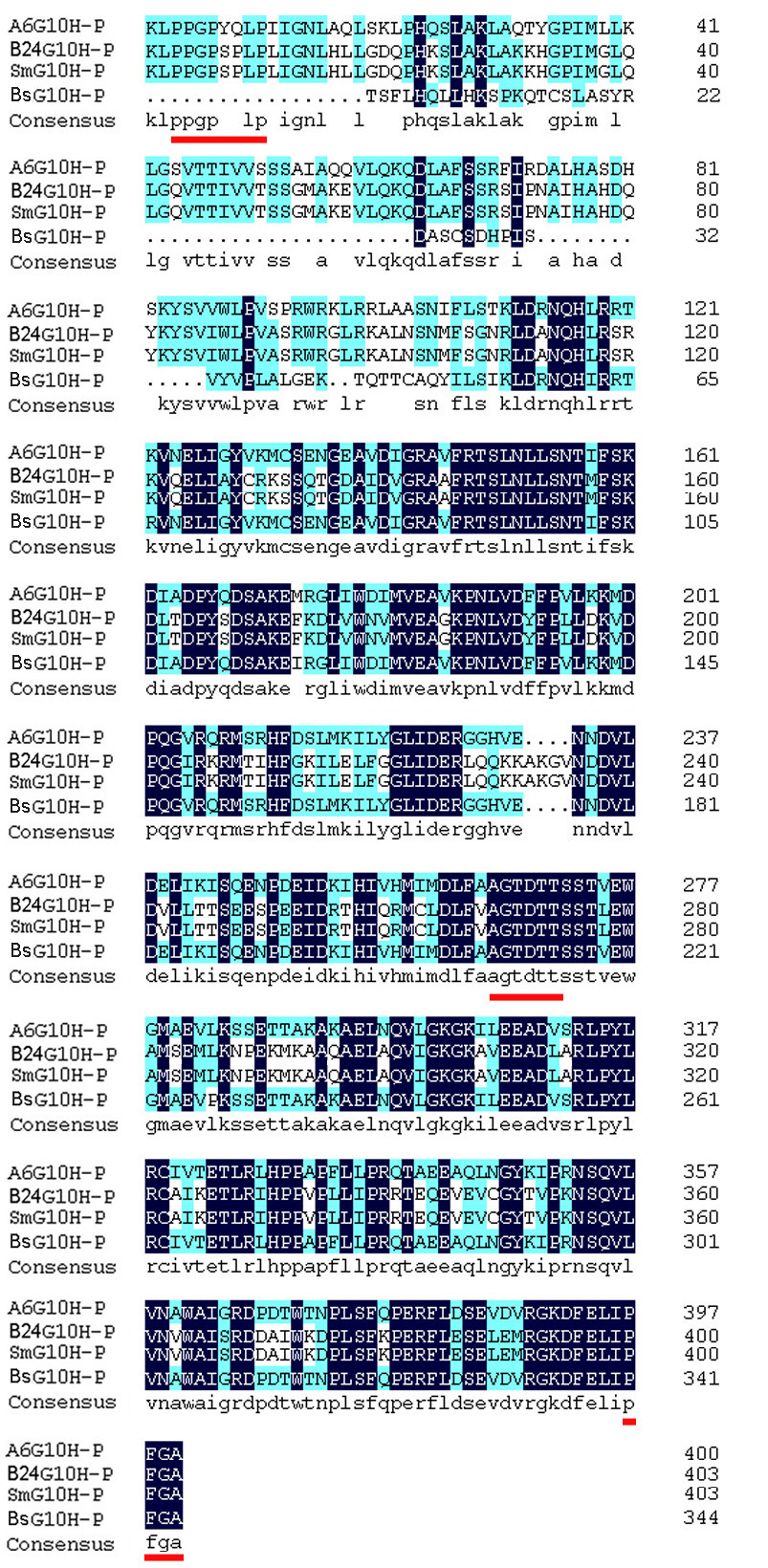

Supplement: Additional file 7 — Alignment of G10H peptide sequences. The red line indicates the conserved domain within the sequence. SmG10H-P, G10H from S. mussotii; B24G10H-P, G10H from hybrid B24; A6G10H-P, G10H from hybrid A6; BsG10H-P, G10H from B. scorzonerifolium. [file 1471-2229-11-71-S7.JPEG]
