# Supplementary material for: Introgression of Swertia mussotii gene into Bupleurum scorzonerifolium via somatic hybridization
Source: BMC Plant Biol. 2011 Apr 25;11:71. doi: 10.1186/1471-2229-11-71 (PMC3098146; doi:10.1186/1471-2229-11-71)
Supplement: Additional file 2 — RAPD analysis of biparental and hybrid calli. Sm: Fragments inherited from S. mussotii; Bs: Fragments inherited from B. scorzonerifolium; T, total of the parents and new bands; N: fragments not present in either biparental profile. [file 1471-2229-11-71-S2.DOC]

Additional file 2. RAPD analysis of biparental and hybrid calli. Sm: Fragments inherited from *S. mussotii*; Bs: Fragments inherited from *B. scorzonerifolium*; T, total of the parents and new bands; N: fragments not present in either biparental profile.

| Primers | Bs | Sm | Hybrid B9 | | | | Hybrid B24 | | | | Hybrid B52 | | | | Hybrid C59 | | | | Hybrid C10 | | | |
| --- | --- | --- | --- | --- | --- | --- | --- | --- | --- | --- | --- | --- | --- | --- | --- | --- | --- | --- | --- | --- | --- | --- |
|  |  |  | T | Bs | Sm | N | T | Bs | Sm | N | T | Bs | Sm | N | T | Bs | Sm | N | T | Bs | Sm | N |
| P04 | 6 | 5 | 6 | 6 | 0 | 0 | 6 | 6 | 0 | 0 | 6 | 6 | 0 | 0 | 6 | 6 | 0 | 0 | 7 | 6 | 0 | 1 |
| P05 | 7 | 6 | 7 | 7 | 0 | 0 | 7 | 7 | 0 | 0 | 8 | 7 | 0 | 1 | 7 | 7 | 0 | 0 | 8 | 7 | 0 | 1 |
| P02 | 6 | 3 | 6 | 6 | 0 | 0 | 6 | 6 | 0 | 0 | 6 | 6 | 0 | 0 | 6 | 6 | 0 | 0 | 5 | 4 | 1 | 0 |
| P03 | 12 | 6 | 12 | 12 | 0 | 0 | 12 | 12 | 0 | 0 | 12 | 12 | 0 | 0 | 12 | 12 | 0 | 0 | 12 | 12 | 0 | 0 |
| P06 | 11 | 7 | 11 | 11 | 0 | 0 | 10 | 10 | 0 | 0 | 10 | 10 | 0 | 0 | 11 | 11 | 0 | 0 | 12 | 11 | 1 | 0 |
| P07 | 4 | 2 | 4 | 4 | 0 | 0 | 4 | 4 | 0 | 0 | 4 | 4 | 0 | 0 | 4 | 4 | 0 | 0 | 3 | 2 | 0 | 1 |
| P08 | 8 | 8 | 8 | 8 | 0 | 0 | 8 | 8 | 0 | 0 | 8 | 8 | 0 | 0 | 8 | 8 | 0 | 0 | 8 | 8 | 0 | 0 |
| P09 | 10 | 8 | 10 | 10 | 0 | 0 | 10 | 10 | 0 | 0 | 10 | 10 | 0 | 0 | 10 | 10 | 0 | 0 | 10 | 9 | 0 | 1 |
| P10 | 4 | 9 | 4 | 4 | 0 | 0 | 4 | 4 | 0 | 0 | 4 | 4 | 0 | 0 | 4 | 4 | 0 | 0 | 4 | 4 | 0 | 0 |
| Q01 | 5 | 4 | 5 | 5 | 0 | 0 | 5 | 5 | 0 | 0 | 5 | 5 | 0 | 0 | 5 | 5 | 0 | 0 | 5 | 5 | 0 | 0 |
| Q03 | 5 | 4 | 5 | 5 | 0 | 0 | 5 | 5 | 0 | 0 | 5 | 5 | 0 | 0 | 5 | 5 | 0 | 0 | 5 | 5 | 0 | 0 |
| Q04 | 5 | 3 | 5 | 5 | 0 | 0 | 5 | 5 | 0 | 0 | 4 | 3 | 1 | 0 | 5 | 5 | 0 | 0 | 5 | 3 | 1 | 1 |
| Q05 | 5 | 10 | 5 | 5 | 0 | 0 | 5 | 5 | 0 | 0 | 5 | 5 | 0 | 0 | 5 | 5 | 0 | 0 | 4 | 4 | 0 | 0 |
| Q06 | 8 | 6 | 8 | 8 | 0 | 0 | 8 | 8 | 0 | 0 | 8 | 8 | 0 | 0 | 8 | 8 | 0 | 0 | 8 | 8 | 0 | 0 |
| Q07 | 9 | 7 | 9 | 9 | 0 | 0 | 9 | 9 | 0 | 0 | 9 | 9 | 0 | 0 | 9 | 9 | 0 | 0 | 9 | 9 | 0 | 0 |
| Q08 | 8 | 8 | 8 | 7 | 0 | 1 | 8 | 8 | 0 | 0 | 8 | 8 | 0 | 0 | 8 | 8 | 0 | 0 | 9 | 5 | 1 | 3 |
| Q09 | 5 | 1 | 5 | 5 | 0 | 0 | 5 | 5 | 0 | 0 | 4 | 3 | 0 | 1 | 5 | 5 | 0 | 0 | 5 | 5 | 0 | 0 |
| Q11 | 7 | 0 | 7 | 7 | 0 | 0 | 7 | 7 | 0 | 0 | 7 | 7 | 0 | 0 | 7 | 7 | 0 | 0 | 8 | 7 | 0 | 1 |
| Q12 | 4 | 6 | 4 | 4 | 0 | 0 | 4 | 4 | 0 | 0 | 4 | 4 | 0 | 0 | 4 | 4 | 0 | 0 | 4 | 4 | 0 | 0 |
| Q13 | 6 | 4 | 6 | 6 | 0 | 0 | 6 | 6 | 0 | 0 | 6 | 6 | 0 | 0 | 6 | 6 | 0 | 0 | 6 | 6 | 0 | 0 |
| Q14 | 5 | 11 | 5 | 5 | 0 | 0 | 5 | 5 | 0 | 0 | 5 | 5 | 0 | 0 | 6 | 5 | 0 | 1 | 5 | 5 | 0 | 0 |
| Q15 | 8 | 5 | 8 | 8 | 0 | 0 | 9 | 8 | 1 | 0 | 8 | 8 | 0 | 0 | 8 | 8 | 0 | 0 | 9 | 8 | 1 | 0 |
| Q16 | 7 | 5 | 7 | 7 | 0 | 0 | 7 | 7 | 0 | 0 | 7 | 7 | 0 | 0 | 7 | 7 | 0 | 0 | 7 | 7 | 0 | 0 |
| Q17 | 5 | 5 | 5 | 5 | 0 | 0 | 5 | 5 | 0 | 0 | 5 | 5 | 0 | 0 | 5 | 5 | 0 | 0 | 5 | 5 | 0 | 0 |
| Q18 | 9 | 8 | 9 | 9 | 0 | 0 | 9 | 9 | 0 | 0 | 9 | 9 | 0 | 0 | 9 | 9 | 0 | 0 | 10 | 8 | 0 | 2 |
| Q19 | 4 | 4 | 5 | 4 | 0 | 1 | 5 | 4 | 0 | 1 | 4 | 4 | 0 | 0 | 5 | 4 | 0 | 1 | 4 | 4 | 0 | 0 |
| Q20 | 7 | 6 | 7 | 7 | 0 | 0 | 7 | 7 | 0 | 0 | 5 | 5 | 0 | 0 | 7 | 7 | 0 | 0 | 7 | 5 | 0 | 2 |
| N2 | 6 | 2 | 6 | 6 | 0 | 0 | 4 | 4 | 0 | 0 | 3 | 3 | 0 | 0 | 4 | 4 | 0 | 0 | 1 | 1 | 0 | 0 |
| N3 | 7 | 0 | 0 | 0 | 0 | 0 | 7 | 7 | 0 | 0 | 7 | 7 | 0 | 0 | 7 | 7 | 0 | 0 | 7 | 7 | 0 | 0 |
| N5 | 8 | 7 | 8 | 8 | 0 | 0 | 8 | 8 | 0 | 0 | 8 | 8 | 0 | 0 | 0 | 0 | 0 | 0 | 9 | 8 | 0 | 1 |
| N7 | 6 | 5 | 6 | 5 | 0 | 1 | 5 | 5 | 0 | 0 | 6 | 5 | 0 | 1 | 5 | 5 | 0 | 0 | 1 | 1 | 0 | 0 |
| N8 | 12 | 11 | 12 | 12 | 0 | 0 | 12 | 12 | 0 | 0 | 12 | 12 | 0 | 0 | 12 | 12 | 0 | 0 | 12 | 12 | 0 | 0 |
| N9 | 8 | 8 | 8 | 8 | 0 | 0 | 8 | 8 | 0 | 0 | 8 | 8 | 0 | 0 | 8 | 8 | 0 | 0 | 8 | 8 | 0 | 0 |
| N10 | 4 | 4 | 4 | 4 | 0 | 0 | 4 | 4 | 0 | 0 | 4 | 4 | 0 | 0 | 4 | 4 | 0 | 0 | 5 | 4 | 0 | 1 |
| N11 | 4 | 2 | 4 | 3 | 0 | 1 | 3 | 3 | 0 | 0 | 3 | 3 | 0 | 0 | 4 | 3 | 1 | 0 | 1 | 1 | 0 | 0 |
| N13 | 3 | 2 | 3 | 3 | 0 | 0 | 3 | 3 | 0 | 0 | 2 | 2 | 0 | 0 | 3 | 3 | 0 | 0 | 2 | 2 | 0 | 0 |
| N14 | 3 | 7 | 3 | 3 | 0 | 0 | 3 | 3 | 0 | 0 | 3 | 3 | 0 | 0 | 4 | 3 | 0 | 1 | 4 | 3 | 0 | 1 |
| N15 | 5 | 5 | 5 | 5 | 0 | 0 | 4 | 4 | 0 | 0 | 4 | 4 | 0 | 0 | 5 | 5 | 0 | 0 | 4 | 4 | 0 | 0 |
| N16 | 7 | 1 | 7 | 7 | 0 | 0 | 0 | 0 | 0 | 0 | 7 | 7 | 0 | 0 | 7 | 7 | 0 | 0 | 7 | 7 | 0 | 0 |
| N17 | 5 | 4 | 5 | 5 | 0 | 0 | 5 | 5 | 0 | 0 | 5 | 5 | 0 | 0 | 6 | 5 | 0 | 1 | 5 | 5 | 0 | 0 |
| N18 | 4 | 5 | 3 | 3 | 0 | 0 | 3 | 3 | 0 | 0 | 3 | 3 | 0 | 0 | 6 | 4 | 1 | 1 | 6 | 4 | 1 | 1 |
| N20 | 9 | 7 | 7 | 7 | 0 | 0 | 6 | 6 | 0 | 0 | 6 | 6 | 0 | 0 | 8 | 8 | 0 | 0 | 8 | 8 | 0 | 0 |
| O1 | 6 | 7 | 6 | 6 | 0 | 0 | 6 | 6 | 0 | 0 | 6 | 6 | 0 | 0 | 6 | 6 | 0 | 0 | 6 | 6 | 0 | 0 |
| O2 | 7 | 6 | 7 | 7 | 0 | 0 | 7 | 7 | 0 | 0 | 6 | 6 | 0 | 0 | 7 | 7 | 0 | 0 | 6 | 5 | 0 | 1 |
| O3 | 4 | 1 | 4 | 4 | 0 | 0 | 4 | 4 | 0 | 0 | 4 | 4 | 0 | 0 | 4 | 4 | 0 | 0 | 4 | 4 | 0 | 0 |
| O4 | 6 | 6 | 6 | 6 | 0 | 0 | 6 | 6 | 0 | 0 | 6 | 6 | 0 | 0 | 6 | 6 | 0 | 0 | 6 | 6 | 0 | 0 |
| O5 | 6 | 7 | 6 | 6 | 0 | 0 | 6 | 6 | 0 | 0 | 6 | 6 | 0 | 0 | 6 | 6 | 0 | 0 | 6 | 5 | 0 | 1 |
| O6 | 4 | 4 | 4 | 4 | 0 | 0 | 4 | 4 | 0 | 0 | 4 | 4 | 0 | 0 | 4 | 4 | 0 | 0 | 4 | 4 | 0 | 0 |
| O7 | 7 | 4 | 7 | 7 | 0 | 0 | 7 | 7 | 0 | 0 | 7 | 7 | 0 | 0 | 0 | 0 | 0 | 0 | 7 | 7 | 0 | 0 |
| O8 | 6 | 5 | 6 | 6 | 0 | 0 | 6 | 6 | 0 | 0 | 6 | 6 | 0 | 0 | 6 | 6 | 0 | 0 | 3 | 3 | 0 | 0 |
| O11 | 5 | 4 | 5 | 5 | 0 | 0 | 5 | 5 | 0 | 0 | 5 | 5 | 0 | 0 | 5 | 5 | 0 | 0 | 6 | 5 | 0 | 1 |
| O12 | 6 | 5 | 6 | 6 | 0 | 0 | 6 | 6 | 0 | 0 | 6 | 6 | 0 | 0 | 6 | 6 | 0 | 0 | 7 | 6 | 0 | 1 |
| O13 | 4 | 3 | 3 | 3 | 0 | 0 | 3 | 3 | 0 | 0 | 3 | 3 | 0 | 0 | 3 | 3 | 0 | 0 | 2 | 2 | 0 | 0 |
| M3 | 5 | 6 | 5 | 5 | 0 | 0 | 5 | 5 | 0 | 0 | 5 | 5 | 0 | 0 | 5 | 5 | 0 | 0 | 5 | 5 | 0 | 0 |
| M4 | 6 | 5 | 6 | 6 | 0 | 0 | 6 | 6 | 0 | 0 | 6 | 6 | 0 | 0 | 6 | 6 | 0 | 0 | 7 | 6 | 0 | 1 |
| M7 | 5 | 7 | 6 | 5 | 1 | 0 | 5 | 5 | 0 | 0 | 6 | 5 | 1 | 0 | 5 | 5 | 0 | 0 | 5 | 5 | 0 | 0 |
| M9 | 6 | 6 | 6 | 6 | 0 | 0 | 6 | 6 | 0 | 0 | 6 | 6 | 0 | 0 | 6 | 6 | 0 | 0 | 6 | 6 | 0 | 0 |
| M10 | 4 | 5 | 4 | 4 | 0 | 0 | 4 | 4 | 0 | 0 | 4 | 4 | 0 | 0 | 4 | 4 | 0 | 0 | 6 | 3 | 1 | 2 |
| M12 | 6 | 5 | 6 | 6 | 0 | 0 | 6 | 6 | 0 | 0 | 6 | 6 | 0 | 0 | 6 | 6 | 0 | 0 | 6 | 6 | 0 | 0 |
| M13 | 5 | 6 | 5 | 4 | 1 | 0 | 5 | 5 | 0 | 0 | 5 | 4 | 1 | 0 | 5 | 5 | 0 | 0 | 5 | 4 | 1 | 0 |
| M14 | 7 | 5 | 7 | 7 | 0 | 0 | 7 | 7 | 0 | 0 | 7 | 7 | 0 | 0 | 7 | 7 | 0 | 0 | 7 | 7 | 0 | 0 |
| M15 | 7 | 3 | 7 | 7 | 0 | 0 | 7 | 7 | 0 | 0 | 7 | 7 | 0 | 0 | 7 | 7 | 0 | 0 | 7 | 7 | 0 | 0 |
| M16 | 6 | 3 | 6 | 6 | 0 | 0 | 6 | 6 | 0 | 0 | 6 | 6 | 0 | 0 | 6 | 6 | 0 | 0 | 6 | 6 | 0 | 0 |
| M17 | 9 | 5 | 9 | 9 | 0 | 0 | 9 | 9 | 0 | 0 | 9 | 9 | 0 | 0 | 9 | 9 | 0 | 0 | 10 | 9 | 0 | 1 |
| M18 | 8 | 6 | 8 | 7 | 0 | 1 | 8 | 8 | 0 | 0 | 8 | 8 | 0 | 0 | 8 | 8 | 0 | 0 | 8 | 8 | 0 | 0 |
| T5 | 5 | 6 | 5 | 5 | 0 | 0 | 5 | 5 | 0 | 0 | 5 | 4 | 1 | 0 | 5 | 5 | 0 | 0 | 5 | 5 | 0 | 0 |
| T6 | 6 | 8 | 7 | 6 | 0 | 1 | 6 | 6 | 0 | 0 | 5 | 5 | 0 | 0 | 5 | 5 | 0 | 0 | 5 | 5 | 0 | 0 |
| T7 | 7 | 7 | 5 | 5 | 0 | 0 | 2 | 2 | 0 | 0 | 5 | 5 | 0 | 0 | 5 | 5 | 0 | 0 | 8 | 6 | 1 | 1 |
| T8 | 8 | 6 | 8 | 8 | 0 | 0 | 8 | 8 | 0 | 0 | 8 | 8 | 0 | 0 | 8 | 8 | 0 | 0 | 6 | 6 | 0 | 0 |
| T13 | 6 | 5 | 6 | 6 | 0 | 0 | 6 | 6 | 0 | 0 | 6 | 6 | 0 | 0 | 6 | 6 | 0 | 0 | 6 | 6 | 0 | 0 |
| T14 | 7 | 8 | 7 | 7 | 0 | 0 | 7 | 7 | 0 | 0 | 7 | 7 | 0 | 0 | 7 | 7 | 0 | 0 | 6 | 5 | 1 | 0 |
| T15 | 10 | 7 | 10 | 10 | 0 | 0 | 10 | 10 | 0 | 0 | 10 | 10 | 0 | 0 | 10 | 10 | 0 | 0 | 10 | 10 | 0 | 0 |
| T16 | 6 | 7 | 6 | 6 | 0 | 0 | 6 | 6 | 0 | 0 | 7 | 6 | 0 | 1 | 6 | 6 | 0 | 0 | 7 | 6 | 1 | 0 |
| U3 | 6 | 4 | 6 | 5 | 1 | 0 | 6 | 6 | 0 | 0 | 6 | 6 | 0 | 0 | 6 | 6 | 0 | 0 | 6 | 6 | 0 | 0 |
| U10 | 8 | 4 | 8 | 8 | 0 | 0 | 8 | 8 | 0 | 0 | 8 | 8 | 0 | 0 | 8 | 8 | 0 | 0 | 8 | 8 | 0 | 0 |
| U13 | 6 | 4 | 6 | 6 | 0 | 0 | 6 | 6 | 0 | 0 | 6 | 6 | 0 | 0 | 6 | 6 | 0 | 0 | 6 | 6 | 0 | 0 |
| U15 | 8 | 7 | 8 | 7 | 0 | 1 | 8 | 8 | 0 | 0 | 8 | 8 | 0 | 0 | 8 | 8 | 0 | 0 | 8 | 8 | 0 | 0 |
| U19 | 6 | 5 | 5 | 5 | 0 | 0 | 6 | 6 | 0 | 0 | 6 | 6 | 0 | 0 | 6 | 6 | 0 | 0 | 6 | 5 | 0 | 1 |
| U20 | 5 | 5 | 5 | 5 | 0 | 0 | 5 | 5 | 0 | 0 | 5 | 5 | 0 | 0 | 5 | 5 | 0 | 0 | 5 | 5 | 0 | 0 |
| W4 | 5 | 4 | 6 | 5 | 0 | 1 | 5 | 5 | 0 | 0 | 6 | 5 | 0 | 1 | 5 | 5 | 0 | 0 | 5 | 5 | 0 | 0 |
| W5 | 5 | 4 | 5 | 5 | 0 | 0 | 5 | 5 | 0 | 0 | 5 | 5 | 0 | 0 | 5 | 5 | 0 | 0 | 6 | 5 | 0 | 1 |
| W6 | 5 | 6 | 6 | 5 | 0 | 1 | 5 | 5 | 0 | 0 | 5 | 5 | 0 | 0 | 5 | 5 | 0 | 0 | 5 | 5 | 0 | 0 |
| W7 | 7 | 5 | 7 | 6 | 0 | 1 | 7 | 7 | 0 | 0 | 7 | 7 | 0 | 0 | 6 | 6 | 0 | 0 | 7 | 6 | 0 | 1 |
| W11 | 6 | 3 | 7 | 6 | 0 | 1 | 8 | 6 | 0 | 2 | 2 | 2 | 0 | 0 | 6 | 6 | 0 | 0 | 6 | 6 | 0 | 0 |
| W13 | 5 | 6 | 5 | 5 | 0 | 0 | 5 | 5 | 0 | 0 | 5 | 5 | 0 | 0 | 5 | 5 | 0 | 0 | 5 | 5 | 0 | 0 |
| H19 | 5 | 4 | 5 | 5 | 0 | 0 | 5 | 5 | 0 | 0 | 5 | 5 | 0 | 0 | 5 | 5 | 0 | 0 | 6 | 5 | 1 | 0 |
| A19 | 8 | 6 | 8 | 8 | 0 | 0 | 8 | 8 | 0 | 0 | 8 | 8 | 0 | 0 | 8 | 8 | 0 | 0 | 8 | 8 | 0 | 0 |
| Total | 546 | 456 | 538 | 524 | 3 | 11 | 527 | 523 | 1 | 3 | 526 | 517 | 4 | 5 | 528 | 521 | 2 | 5 | 539 | 498 | 12 | 29 |
|  | Bs | Sm | T | Bs | Sm | N | T | Bs | Sm | N | T | Bs | Sm | N | T | Bs | Sm | T | T | Bs | Sm | N |
