# Supplementary material for: Introgression of Swertia mussotii gene into Bupleurum scorzonerifolium via somatic hybridization
Source: BMC Plant Biol. 2011 Apr 25;11:71. doi: 10.1186/1471-2229-11-71 (PMC3098146; doi:10.1186/1471-2229-11-71)
Supplement: Additional file 3 — Frequency in the hybrid clones of donor fragments, and fragments absent from both biparental profiles. [file 1471-2229-11-71-S3.DOC]

Additional file 3. Frequency in the hybrid clones of donor fragments, and fragments absent from both biparental profiles.

|  | Total bands | Donor bands | New bands | Donor bands/  Total bands (%) | New bands/  Total bands (%) |
| --- | --- | --- | --- | --- | --- |
| Hybrid B9  (UV30s) | 538 | 3 | 11 | 0.6 | 2.0 |
| Hybrid B24  (UV30s) | 527 | 1 | 3 | 0.2 | 0.6 |
| Hybrid B52  (UV30s) | 526 | 4 | 5 | 0.8 | 1.0 |
| Hybrid C59  (UV60s) | 528 | 2 | 5 | 0.4 | 1.0 |
| Hybrid C10  (UV60s) | 539 | 12 | 29 | 2.4 | 5.4 |
