# Supplementary material for: Introgression of Swertia mussotii gene into Bupleurum scorzonerifolium via somatic hybridization
Source: BMC Plant Biol. 2011 Apr 25;11:71. doi: 10.1186/1471-2229-11-71 (PMC3098146; doi:10.1186/1471-2229-11-71)
Supplement: Additional file 4 — GC-MS analysis of volatile compounds present in the biparental and hybrid calli. [file 1471-2229-11-71-S4.DOC]

Additional file 4. GC-MS analysis of volatile compounds present in the biparental and hybrid calli.

| Sample | Compound | Molecular formula | *S. mussotii* | Hybrid  A6 | Hybrid B24 | *B. scorzonerifolium* |
| --- | --- | --- | --- | --- | --- | --- |
| 1 | ethanedioic acid | C_2_H_2_O_4_ | + | + | + | + |
| 2 | acetic acid | C_2_H_4_O_2_ | + | + | + | + |
| 3 | propionaldehyde | C_3_H_6_O | + | + | + | + |
| 4 | 3-H- pyrazole -3 -ketone | C_3_H_4_N_2_O | + | - | - | + |
| 5 | 2-(hydroxymethyl)-2-nitro-1,3-propanediol | C_4_H_9_NO_5_ | + | + | + | + |
| 6 | 2-pyrrolidone | C_4_H_7_NO | - | - | + | + |
| 7 | diethylnitrosamine | C_4_H_10_N_2_O | + | - | + | + |
| 8 | 2- furaldehyde | C_5_H_3_O_2_ | + | + | - | + |
| 9 | 4-H- pyran | C_5_H_6_O | + | - | - | - |
| 10 | furfuryl alcohol | C_5_H_6_O_2_ | + | - | - | + |
| 11 | 1,2-cyclopentanedione | C_5_H_6_O_2_ | - | - | - | + |
| 12 | 2-aminopyridine | C_5_H_6_N_2_ | + | **-** | **-** | - |
| 13 | dihydropyran | C_5_H_8_O | + | - | + | - |
| 14 | 2-hydroxy-2-methylbutanedioic acid | C_5_H_8_O_5_ | + | - | - | + |
| 15 | 2- methylbutyric acid | C_5_H_10_O_2_ | + | - | - | + |
| 16 | 2-amino-1-pentanol | C_5_H_13_NO | - | + | + | + |
| 17 | L-ornithine Hydrochloride | C_5_H_12_N_2_O_2_ | + | + | + | + |
| 18 | 2,3 -dimethyl pyrazine | C_6_H_8_N_2_ | + | - | + | - |
| 19 | 4- methyl-5- alcohol thiazole | C_6_H_8_NSO | + | - | - | + |
| 20 | cyclohexanol | C_6_H_12_O | - | + | + | - |
| 21 | 2,4-dihydroxy-2,5-dimethyl-3(2H)-furanone | C_6_H_8_O_4_ | + | + | - | - |
| 22 | 2,5- methenyl furfuran | C_6_H_3_O_3_ | - | + | + | - |
| 23 | dioxolane | C_6_H_12_O_6_ | + | - | - | - |
| 24 | 2-hydroxymethylfurfural | C_6_H_6_O_3_ | + | - | - | - |
| 25 | 3- pyrazolidone | C_6_H_10_N_2_O | + | - | - | - |
| 26 | 2,5-dimethyl-4-hydroxy-3(2H)-furanone | C_6_H_8_O_3_ | - | + | - | - |
| 27 | ethyl vinyl ether | C_6_H_12_O | - | - | + | - |
| 28 | caprolactam | C_6_H_11_NO | + | - | - | - |
| 29 | phenol | C_6_H_6_O | + | + | + | - |
| 30 | heptylic acid | C_7_H_14_O_2_ | + | - | + | - |
| 31 | diethyl cyclopentane | C_7_H_20_ | - | + | + | + |
| 32 | 1-(3-aminopropoxy)-2-ethoxyethane | C_7_H_17_NO_2_ | + | - | + | - |
| 33 | glyceraldehyde dipolymer | C_7_H_17_NO_2_ | + | **-** | **-** | + |
| 34 | benzyl alcohol | C_7_H_8_O | + | - | - | - |
| 35 | salicylic acid | C_7_H_6_O_3_ | + | + | + | - |
| 35 | benzoic acid | C_7_H_6_O_2_ | + | - | - | - |
| 36 | hyacinthin | C_8_H_8_O | - | - | + | + |
| 37 | 2,5-dimethyl-4-hydroxy-3-furanone | C_8_H_14_O_3_ | - | + | - | + |
| 38 | indole | C_8_H_7_N | + | - | + | + |
| 39 | benzendicarboxylic acid | C_8_H_6_O_4_ | + | - | + | - |
| 40 | coumaron | C_8_H_8_O | + | - | + | - |
| 41 | phenylacetaldehyde | C_8_H_8_O | + | - | - | - |
| 42 | 2-methyl -4 –vinylphenol | C_9_H_10_O_2_ | + | - | - | - |
| 43 | octane | C_10_H_22_ | + | + | + | + |
| 44 | diethyl -3,3’- oxydipropionitrile | C_10_H_18_N_2_O | + | - | - | - |
| 45 | methylnaphthalene | C_11_H_10_ | - | - | + | + |
| 46 | 6- dodecanone | C_12_H_18_O | + | - | - | - |
| 47 | dodecanoyl | C_12_H_24_O | - | + | + | - |
| 48 | asaricin | C_12_H_16_O_3_ | + | + | + | + |
| 49 | 2,5- dimethyl hendecane | C_13_H_28_ | - | - | - | + |
| 50 | tetradecanoic acid | C_14_H_28_O_2_ | - | + | - | + |
| 51 | tetradecanol | C_14_H_28_O | + | - | + | + |
| 52 | cyclohexene methyl caffeine | C_15_H_20_N_4_O_2_ | + | - | - | - |
| 53 | 1-hydroxy-3,7,8-trimethoxyxanthenone | C_16_H_14_O_6_ | + | - | + | - |
| 54 | ethyl myristate | C_16_H_32_O_2_ | + | - | - | + |
| 55 | Sclareolide | C_16_ H_26_O_2_ | + | - | - | - |
| 56 | hexadecanoic acid | C_16_H_32_O_2_ | + | + | + | + |
| 57 | octadecyl triene | C_18_H_32_ | + | - | - | - |
| 58 | linoleic acid | C_18_H_32_O_2_ | + | - | + | - |
| 59 | oleic acid | C_18_H_34_O_2_ | + | + | + | + |
| 60 | stearic acid | C_18_H_36_O_2_ | + | + | + | + |
| 61 | 1,2 benzendicarboxylic acid octyl nitrite | C_24_H_38_O_4_ | + | + | - | - |
| 62 | 2-Ethylhexyl hexadecanoate | C_24_H_48_O_2_ | + | + | - | + |
| 63 | anthropodeoxycholic acid | C_24_H_40_O_4_ | + | - | - | - |
| 64 | stigmasterine | C_29_H_48_O | + | - | - | + |
| 65 | squalene | C_30_H_50_ | + | - | - | - |
