# Supplementary material for: Introgression of Swertia mussotii gene into Bupleurum scorzonerifolium via somatic hybridization
Source: BMC Plant Biol. 2011 Apr 25;11:71. doi: 10.1186/1471-2229-11-71 (PMC3098146; doi:10.1186/1471-2229-11-71)
Supplement: Additional file 5 — Sequences of primer used for these experiments. [file 1471-2229-11-71-S5.DOC]

Additional file 5. Sequences of primer used for these experiments.

| CYP | Primer |
| --- | --- |
| CYP76F | aaamttccwccnggdccat |
| CYP76R | cdgcacckaadggaatcagc |
| CYP51F | atgctagrcatcyaaggcta |
| CYP51R | tcamgcadcgaacggagtga |
| CYP71F | agnaccctggasgcatttcg |
| CYP71R | ttkgagtgngcttggccaaa |
| CYP79F | taytggmrgttymtaagaa |
| CYP79R | ccadtsaagtgdatcatg |
| CYP72F | gtagcntcgtagtnythcggact |
| CYP72R | aarytctccaaryttcatctcgtt |
| CYP90F | ttcnccraanargtgggtca |
| CYP90R | cckccanggattraacgtgct |
